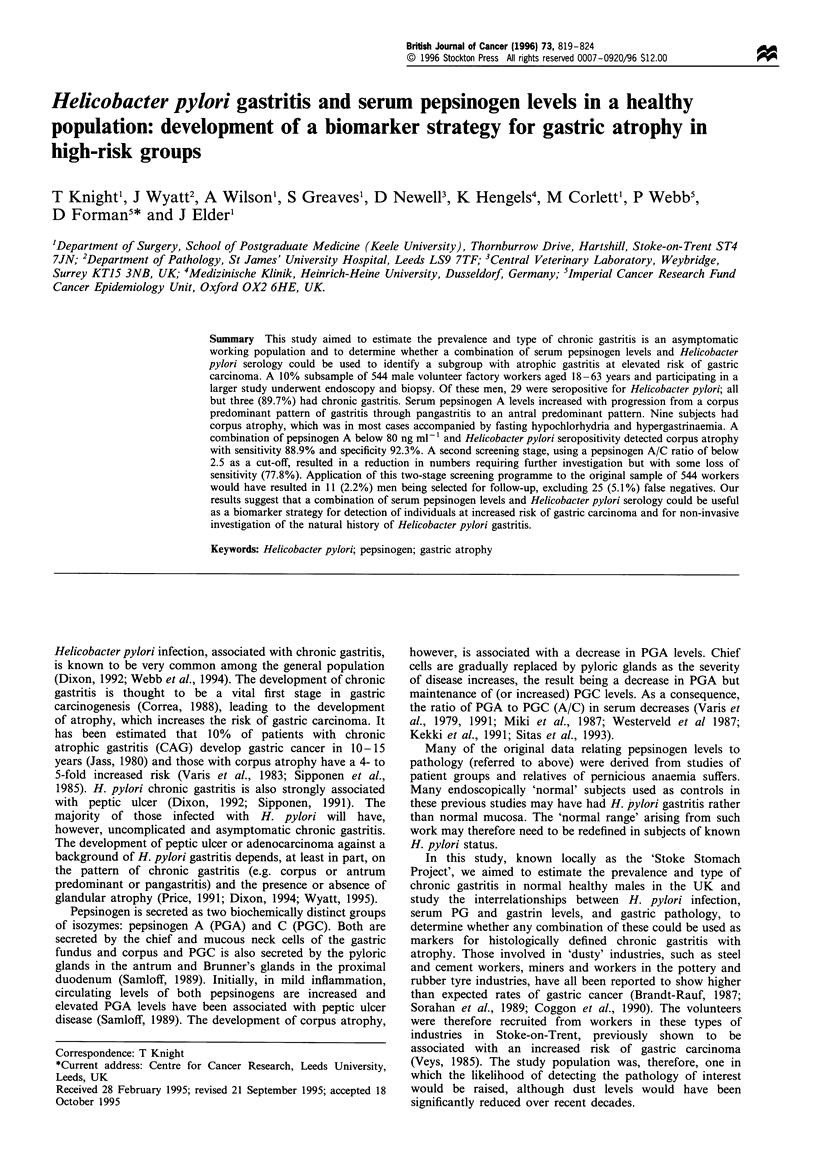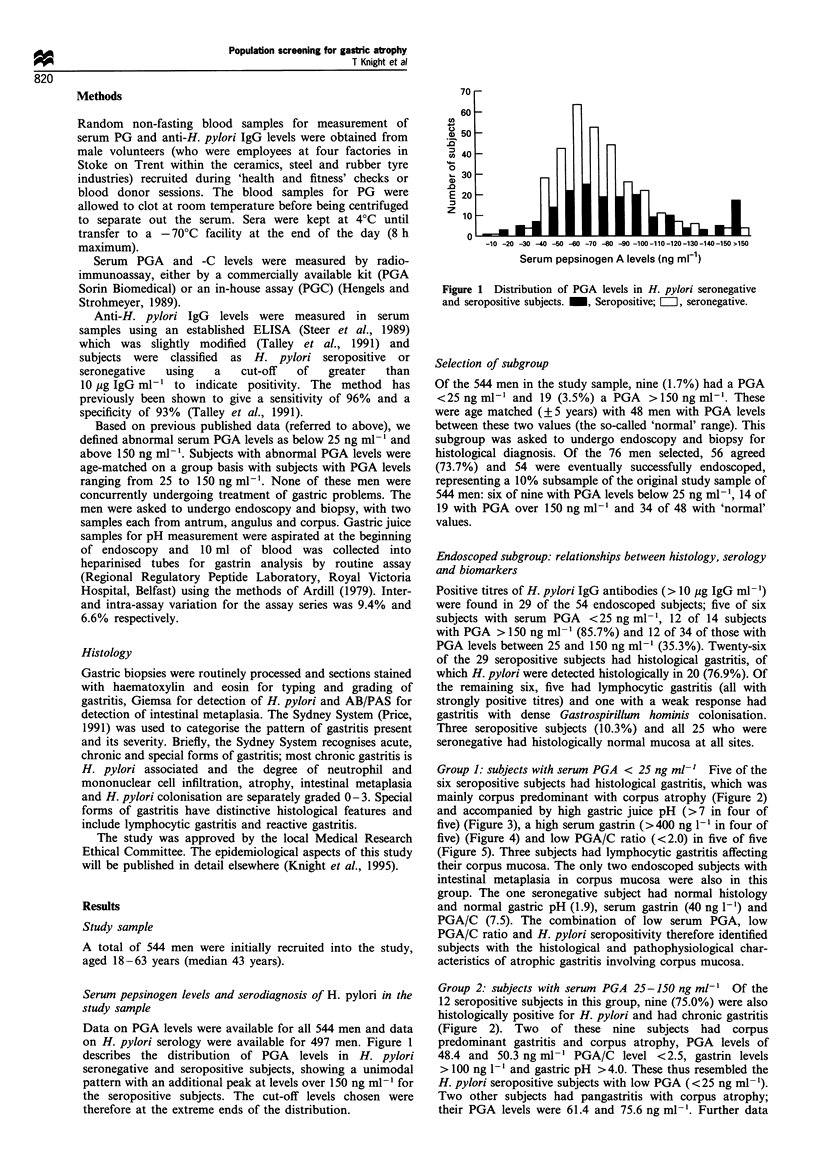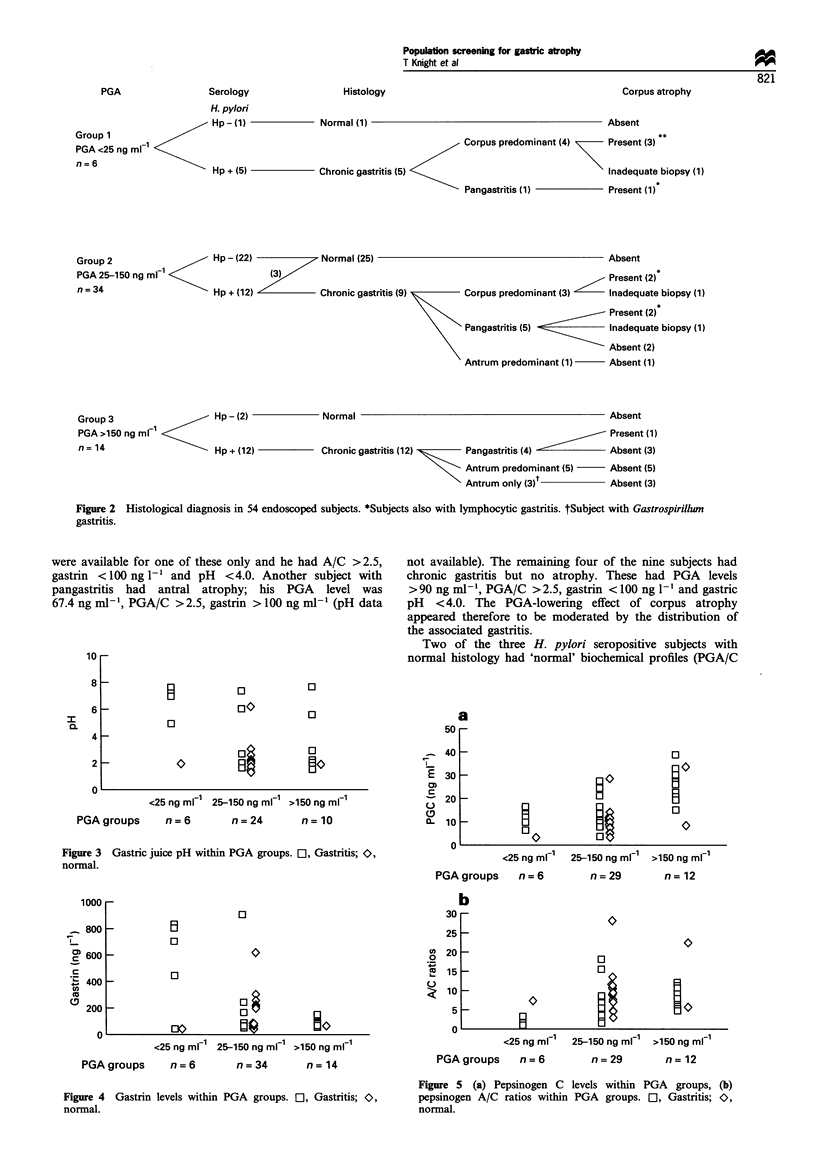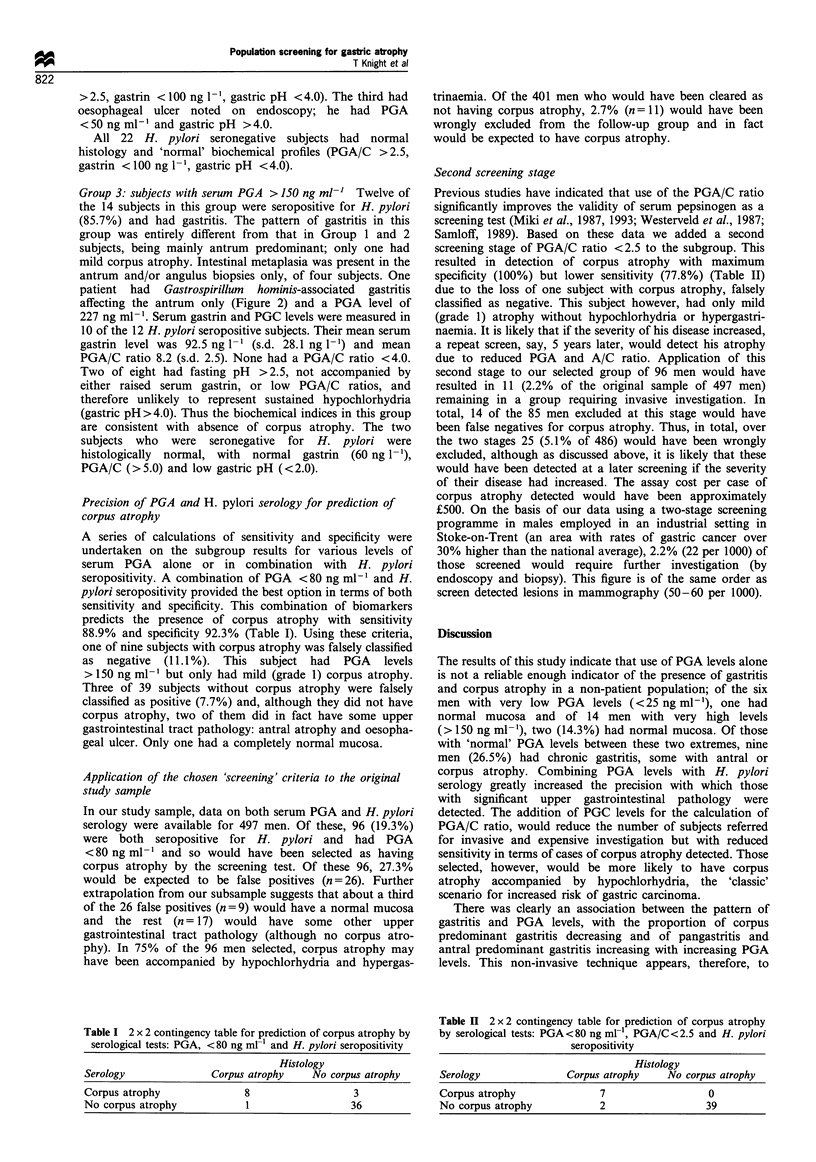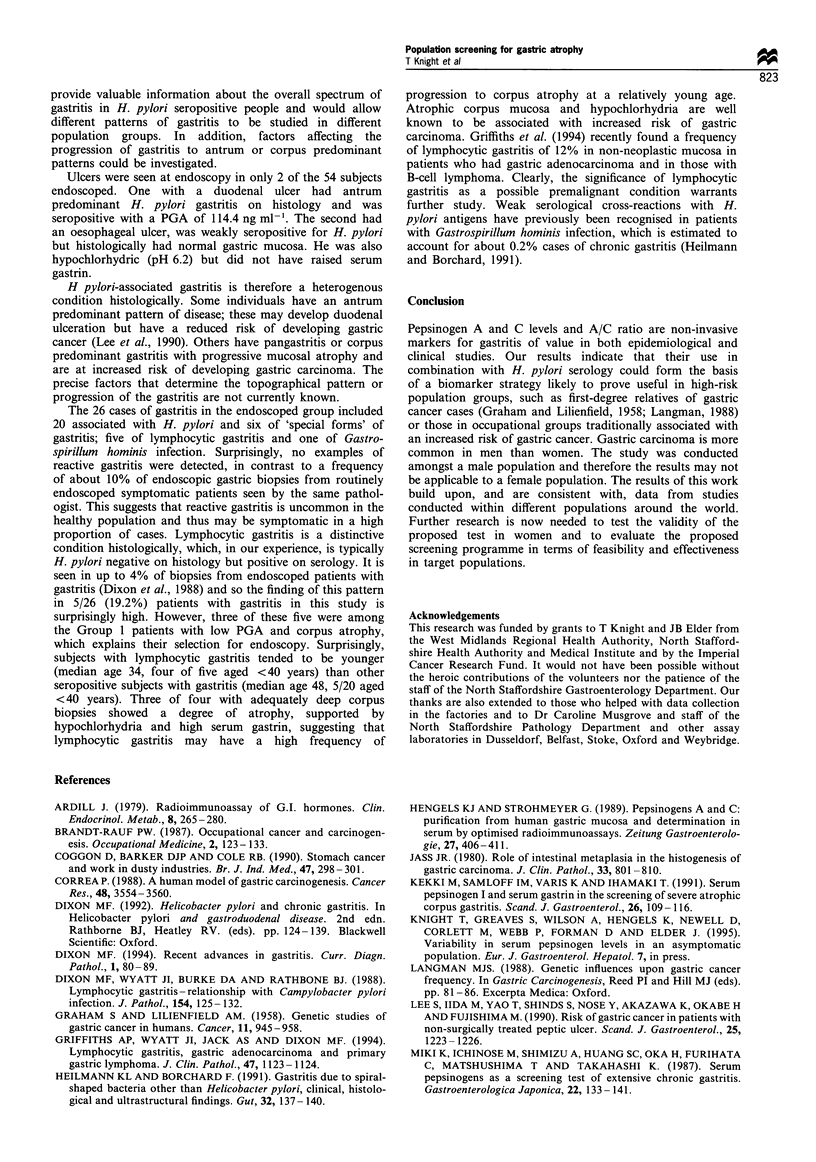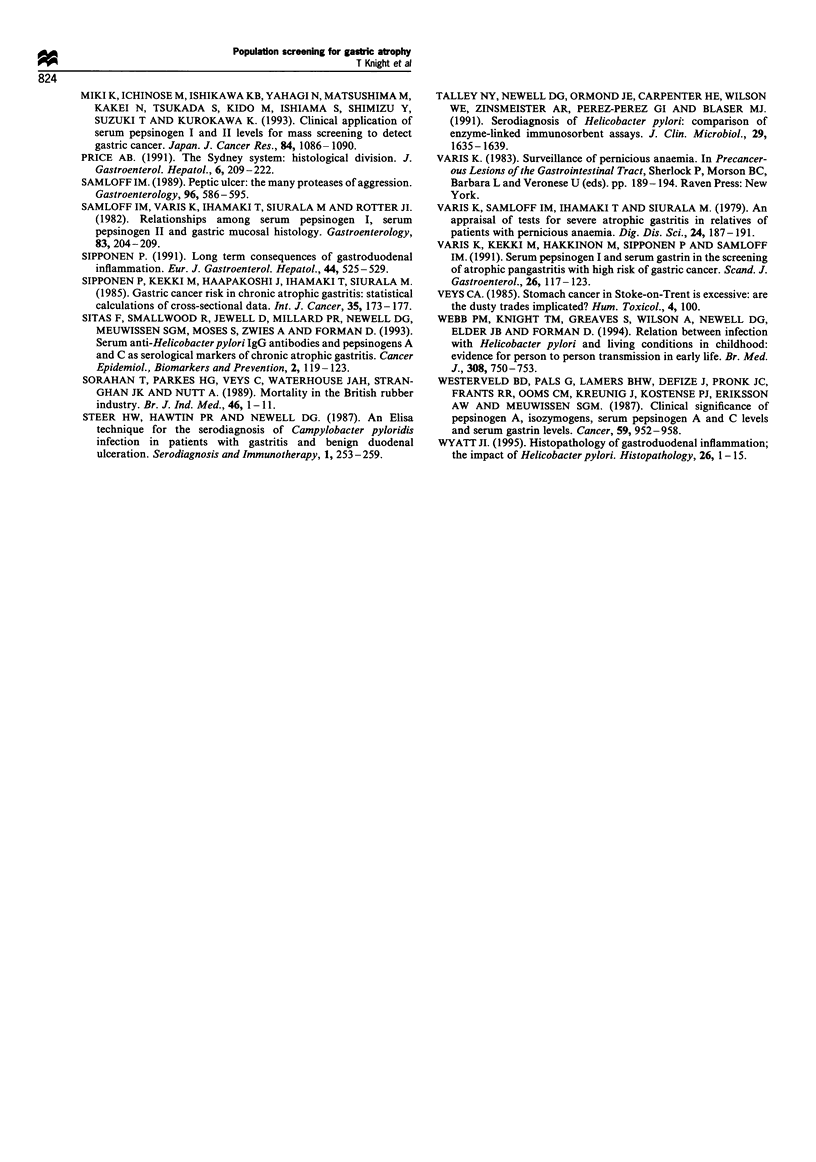# Helicobacter pylori gastritis and serum pepsinogen levels in a healthy population: development of a biomarker strategy for gastric atrophy in high risk groups.

**DOI:** 10.1038/bjc.1996.144

**Published:** 1996-03

**Authors:** T. Knight, J. Wyatt, A. Wilson, S. Greaves, D. Newell, K. Hengels, M. Corlett, P. Webb, D. Forman, J. Elder

**Affiliations:** Depaprtment of Surgery, School of Postgraduate Medicine (Keele University), Stoke-on-Trent, UK.

## Abstract

This study aimed to estimate the prevalence and type of chronic gastritis in an asymptomatic working population and to determine whether a combination of serum pepsinogen levels and Helicobacter pylori serology could be used to identify a subgroup with atrophic gastritis at elevated risk of gastric carcinoma. A 10% subsample of 544 male volunteer factory workers aged 18-63 years and participating in a larger study underwent endoscopy and biopsy. Of these men, 29 were seropositive for Helicobacter pylori; all but three (89.7%) had chronic gastritis. Serum pepsinogen A levels increased with progression from a corpus predominant pattern of gastritis through pangastritis to an antral predominant pattern. Nine subjects had corpus atrophy, which was in most cases accompanied by fasting hypochlorhydria and hypergastrinaemia. A combination of pepsinogen A below 80 ng ml-1 and Helicobaceter pylori seropositivity detected corpus atrophy with sensitivity 88.9% and specificity 92.3%. A second screening stage, using a pepsinogen A/C ratio of below 2.5 as a cut-off, resulted in a reduction in numbers requiring further investigation but with some loss of sensitivity (77.8%). Application of this two-stage screening programme to the original sample of 544 workers would have resulted in 11 (2.2%) men being selected for follow-up, excluding 25 (5.1%) false negatives. Our results suggest that a combination of serum pepsinogen levels and Helicobacter pylori serology could be useful as a biomarker strategy for detection of individuals at increased risk of gastric carcinoma and for non-invasive investigation of the natural history of Helicobacter pylori gastritis.


British Journal of Cancer (1996) 73, 819-824
© 1996 Stockton Press All rights reserved 0007-0920/96 $12.00

### *Helicobacter pylori* gastritis and serum pepsinogen levels in a healthy population: development of a biomarker strategy for gastric atrophy in high-risk groups

T Knight[1], J Wyatt[2], A Wilson[1], S Greaves[1], D Newell[3], K Hengels[4], M Corlett[1], P Webb[5], D Forman[5]* and J Elder[1]

[1]*Department of Surgery, School of Postgraduate Medicine (Keele University), Thornburrow Drive, Hartshill, Stoke-on-Trent ST4 7JN;* [2]*Department of Pathology, St James' University Hospital, Leeds LS9 7TF;* [3]*Central Veterinary Laboratory, Weybridge, Surrey KT15 3NB, UK;* [4]*Medizinische Klinik, Heinrich-Heine University, Dusseldorf, Germany;* [5]*Imperial Cancer Research Fund Cancer Epidemiology Unit, Oxford OX2 6HE, UK.*

**Summary** This study aimed to estimate the prevalence and type of chronic gastritis is an asymptomatic working population and to determine whether a combination of serum pepsinogen levels and *Helicobacter pylori* serology could be used to identify a subgroup with atrophic gastritis at elevated risk of gastric carcinoma. A 10% subsample of 544 male volunteer factory workers aged 18-63 years and participating in a larger study underwent endoscopy and biopsy. Of these men, 29 were seropositive for *Helicobacter pylori*; all but three (89.7%) had chronic gastritis. Serum pepsinogen A levels increased with progression from a corpus predominant pattern of gastritis through pangastritis to an antral predominant pattern. Nine subjects had corpus atrophy, which was in most cases accompanied by fasting hypochlorhydria and hypergastrinaemia. A combination of pepsinogen A below 80 ng $ml^{-1}$ and *Helicobacter pylori* seropositivity detected corpus atrophy with sensitivity 88.9% and specificity 92.3%. A second screening stage, using a pepsinogen A/C ratio of below 2.5 as a cut-off, resulted in a reduction in numbers requiring further investigation but with some loss of sensitivity (77.8%). Application of this two-stage screening programme to the original sample of 544 workers would have resulted in 11 (2.2%) men being selected for follow-up, excluding 25 (5.1%) false negatives. Our results suggest that a combination of serum pepsinogen levels and *Helicobacter pylori* serology could be useful as a biomarker strategy for detection of individuals at increased risk of gastric carcinoma and for non-invasive investigation of the natural history of *Helicobacter pylori* gastritis.

**Keywords:** *Helicobacter pylori*; pepsinogen; gastric atrophy

*Helicobacter pylori* infection, associated with chronic gastritis, is known to be very common among the general population (Dixon, 1992; Webb *et al.*, 1994). The development of chronic gastritis is thought to be a vital first stage in gastric carcinogenesis (Correa, 1988), leading to the development of atrophy, which increases the risk of gastric carcinoma. It has been estimated that 10% of patients with chronic atrophic gastritis (CAG) develop gastric cancer in 10-15 years (Jass, 1980) and those with corpus atrophy have a 4- to 5-fold increased risk (Varis *et al.*, 1983; Sipponen *et al.*, 1985). *H. pylori* chronic gastritis is also strongly associated with peptic ulcer (Dixon, 1992; Sipponen, 1991). The majority of those infected with *H. pylori* will have, however, uncomplicated and asymptomatic chronic gastritis. The development of peptic ulcer or adenocarcinoma against a background of *H. pylori* gastritis depends, at least in part, on the pattern of chronic gastritis (e.g. corpus or antrum predominant or pangastritis) and the presence or absence of glandular atrophy (Price, 1991; Dixon, 1994; Wyatt, 1995).

Pepsinogen is secreted as two biochemically distinct groups of isozymes: pepsinogen A (PGA) and C (PGC). Both are secreted by the chief and mucous neck cells of the gastric fundus and corpus and PGC is also secreted by the pyloric glands in the antrum and Brunner's glands in the proximal duodenum (Samloff, 1989). Initially, in mild inflammation, circulating levels of both pepsinogens are increased and elevated PGA levels have been associated with peptic ulcer disease (Samloff, 1989). The development of corpus atrophy, however, is associated with a decrease in PGA levels. Chief cells are gradually replaced by pyloric glands as the severity of disease increases, the result being a decrease in PGA but maintenance of (or increased) PGC levels. As a consequence, the ratio of PGA to PGC (A/C) in serum decreases (Varis *et al.*, 1979, 1991; Miki *et al.*, 1987; Westerveld *et al* 1987; Kekki *et al.*, 1991; Sitas *et al.*, 1993).

Many of the original data relating pepsinogen levels to pathology (referred to above) were derived from studies of patient groups and relatives of pernicious anaemia suffers. Many endoscopically 'normal' subjects used as controls in these previous studies may have had *H. pylori* gastritis rather than normal mucosa. The 'normal range' arising from such work may therefore need to be redefined in subjects of known *H. pylori* status.

In this study, known locally as the 'Stoke Stomach Project', we aimed to estimate the prevalence and type of chronic gastritis in normal healthy males in the UK and study the interrelationships between *H. pylori* infection, serum PG and gastrin levels, and gastric pathology, to determine whether any combination of these could be used as markers for histologically defined chronic gastritis with atrophy. Those involved in 'dusty' industries, such as steel and cement workers, miners and workers in the pottery and rubber tyre industries, have all been reported to show higher than expected rates of gastric cancer (Brandt-Rauf, 1987; Sorahan *et al.*, 1989; Coggon *et al.*, 1990). The volunteers were therefore recruited from workers in these types of industries in Stoke-on-Trent, previously shown to be associated with an increased risk of gastric carcinoma (Veys, 1985). The study population was, therefore, one in which the likelihood of detecting the pathology of interest would be raised, although dust levels would have been significantly reduced over recent decades.

Correspondence: T Knight
*Current address: Centre for Cancer Research, Leeds University, Leeds, UK
Received 28 February 1995; revised 21 September 1995; accepted 18 October 1995

### Methods

Random non-fasting blood samples for measurement of serum PG and anti-*H. pylori* IgG levels were obtained from male volunteers (who were employees at four factories in Stoke on Trent within the ceramics, steel and rubber tyre industries) recruited during 'health and fitness' checks or blood donor sessions. The blood samples for PG were allowed to clot at room temperature before being centrifuged to separate out the serum. Sera were kept at 4°C until transfer to a −70°C facility at the end of the day (8 h maximum).

Serum PGA and -C levels were measured by radioimmunoassay, either by a commercially available kit (PGA Sorin Biomedical) or an in-house assay (PGC) (Hengels and Strohmeyer, 1989).

Anti-*H. pylori* IgG levels were measured in serum samples using an established ELISA (Steer *et al.*, 1989) which was slightly modified (Talley *et al.*, 1991) and subjects were classified as *H. pylori* seropositive or seronegative using a cut-off of greater than 10 $\mu$g IgG $ml^{-1}$ to indicate positivity. The method has previously been shown to give a sensitivity of 96% and a specificity of 93% (Talley *et al.*, 1991).

Based on previous published data (referred to above), we defined abnormal serum PGA levels as below 25 ng $ml^{-1}$ and above 150 ng $ml^{-1}$. Subjects with abnormal PGA levels were age-matched on a group basis with subjects with PGA levels ranging from 25 to 150 ng $ml^{-1}$. None of these men were concurrently undergoing treatment of gastric problems. The men were asked to undergo endoscopy and biopsy, with two samples each from antrum, angulus and corpus. Gastric juice samples for pH measurement were aspirated at the beginning of endoscopy and 10 ml of blood was collected into heparinised tubes for gastrin analysis by routine assay (Regional Regulatory Peptide Laboratory, Royal Victoria Hospital, Belfast) using the methods of Ardill (1979). Inter- and intra-assay variation for the assay series was 9.4% and 6.6% respectively.

### *Histology*

Gastric biopsies were routinely processed and sections stained with haematoxylin and eosin for typing and grading of gastritis, Giemsa for detection of *H. pylori* and AB/PAS for detection of intestinal metaplasia. The Sydney System (Price, 1991) was used to categorise the pattern of gastritis present and its severity. Briefly, the Sydney System recognises acute, chronic and special forms of gastritis; most chronic gastritis is *H. pylori* associated and the degree of neutrophil and mononuclear cell infiltration, atrophy, intestinal metaplasia and *H. pylori* colonisation are separately graded 0–3. Special forms of gastritis have distinctive histological features and include lymphocytic gastritis and reactive gastritis.

The study was approved by the local Medical Research Ethical Committee. The epidemiological aspects of this study will be published in detail elsewhere (Knight *et al.*, 1995).

### Results

### *Study sample*

A total of 544 men were initially recruited into the study, aged 18–63 years (median 43 years).

### *Serum pepsinogen levels and serodiagnosis of* H. pylori *in the study sample*

Data on PGA levels were available for all 544 men and data on *H. pylori* serology were available for 497 men. Figure 1 describes the distribution of PGA levels in *H. pylori* seronegative and seropositive subjects, showing a unimodal pattern with an additional peak at levels over 150 ng $ml^{-1}$ for the seropositive subjects. The cut-off levels chosen were therefore at the extreme ends of the distribution.

**Figure 1** Distribution of PGA levels in *H. pylori* seronegative and seropositive subjects. ■, Seropositive; □, seronegative.

### *Selection of subgroup*

Of the 544 men in the study sample, nine (1.7%) had a PGA <25 ng $ml^{-1}$ and 19 (3.5%) a PGA >150 ng $ml^{-1}$. These were age matched (±5 years) with 48 men with PGA levels between these two values (the so-called 'normal' range). This subgroup was asked to undergo endoscopy and biopsy for histological diagnosis. Of the 76 men selected, 56 agreed (73.7%) and 54 were eventually successfully endoscoped, representing a 10% subsample of the original study sample of 544 men: six of nine with PGA levels below 25 ng $ml^{-1}$, 14 of 19 with PGA over 150 ng $ml^{-1}$ and 34 of 48 with 'normal' values.

### *Endoscoped subgroup: relationships between histology, serology and biomarkers*

Positive titres of *H. pylori* IgG antibodies (>10 $\mu$g IgG $ml^{-1}$) were found in 29 of the 54 endoscoped subjects; five of six subjects with serum PGA <25 ng $ml^{-1}$, 12 of 14 subjects with PGA >150 ng $ml^{-1}$ (85.7%) and 12 of 34 of those with PGA levels between 25 and 150 ng $ml^{-1}$ (35.3%). Twenty-six of the 29 seropositive subjects had histological gastritis, of which *H. pylori* were detected histologically in 20 (76.9%). Of the remaining six, five had lymphocytic gastritis (all with strongly positive titres) and one with a weak response had gastritis with dense *Gastrospirillum hominis* colonisation. Three seropositive subjects (10.3%) and all 25 who were seronegative had histologically normal mucosa at all sites.

*Group 1: subjects with serum PGA < 25 ng $ml^{-1}$* Five of the six seropositive subjects had histological gastritis, which was mainly corpus predominant with corpus atrophy (Figure 2) and accompanied by high gastric juice pH (>7 in four of five) (Figure 3), a high serum gastrin (>400 ng $l^{-1}$ in four of five) (Figure 4) and low PGA/C ratio (<2.0) in five of five (Figure 5). Three subjects had lymphocytic gastritis affecting their corpus mucosa. The only two endoscoped subjects with intestinal metaplasia in corpus mucosa were also in this group. The one seronegative subject had normal histology and normal gastric pH (1.9), serum gastrin (40 ng $l^{-1}$) and PGA/C (7.5). The combination of low serum PGA, low PGA/C ratio and *H. pylori* seropositivity therefore identified subjects with the histological and pathophysiological characteristics of atrophic gastritis involving corpus mucosa.

*Group 2: subjects with serum PGA 25–150 ng $ml^{-1}$* Of the 12 seropositive subjects in this group, nine (75.0%) were also histologically positive for *H. pylori* and had chronic gastritis (Figure 2). Two of these nine subjects had corpus predominant gastritis and corpus atrophy, PGA levels of 48.4 and 50.3 ng $ml^{-1}$ PGA/C level <2.5, gastrin levels >100 ng $l^{-1}$ and gastric pH >4.0. These thus resembled the *H. pylori* seropositive subjects with low PGA (<25 ng $ml^{-1}$). Two other subjects had pangastritis with corpus atrophy; their PGA levels were 61.4 and 75.6 ng $ml^{-1}$. Further data

**Figure 2** Histological diagnosis in 54 endoscoped subjects. *Subjects also with lymphocytic gastritis. †Subject with *Gastrospirillum* gastritis.

were available for one of these only and he had A/C >2.5, gastrin <100 ng $l^{-1}$ and pH <4.0. Another subject with pangastritis had antral atrophy; his PGA level was 67.4 ng $ml^{-1}$, PGA/C >2.5, gastrin >100 ng $ml^{-1}$ (pH data not available). The remaining four of the nine subjects had chronic gastritis but no atrophy. These had PGA levels >90 ng $ml^{-1}$, PGA/C >2.5, gastrin <100 ng $l^{-1}$ and gastric pH <4.0. The PGA-lowering effect of corpus atrophy appeared therefore to be moderated by the distribution of the associated gastritis.

Two of the three *H. pylori* seropositive subjects with normal histology had 'normal' biochemical profiles (PGA/C

**Figure 3** Gastric juice pH within PGA groups. □, Gastritis; ◇, normal.

**Figure 4** Gastrin levels within PGA groups. □, Gastritis; ◇, normal.

**Figure 5** (a) Pepsinogen C levels within PGA groups, (b) pepsinogen A/C ratios within PGA groups. □, Gastritis; ◇, normal.

>2.5, gastrin <100 ng $l^{-1}$, gastric pH <4.0). The third had oesophageal ulcer noted on endoscopy; he had PGA <50 ng $ml^{-1}$ and gastric pH >4.0.

All 22 *H. pylori* seronegative subjects had normal histology and 'normal' biochemical profiles (PGA/C >2.5, gastrin <100 ng $l^{-1}$, gastric pH <4.0).

*Group 3: subjects with serum PGA >150 ng $ml^{-1}$* Twelve of the 14 subjects in this group were seropositive for *H. pylori* (85.7%) and had gastritis. The pattern of gastritis in this group was entirely different from that in Group 1 and 2 subjects, being mainly antrum predominant; only one had mild corpus atrophy. Intestinal metaplasia was present in the antrum and/or angulus biopsies only, of four subjects. One patient had *Gastrospirillum hominis*-associated gastritis affecting the antrum only (Figure 2) and a PGA level of 227 ng $ml^{-1}$. Serum gastrin and PGC levels were measured in 10 of the 12 *H. pylori* seropositive subjects. Their mean serum gastrin level was 92.5 ng $l^{-1}$ (s.d. 28.1 ng $l^{-1}$) and mean PGA/C ratio 8.2 (s.d. 2.5). None had a PGA/C ratio <4.0. Two of eight had fasting pH >2.5, not accompanied by either raised serum gastrin, or low PGA/C ratios, and therefore unlikely to represent sustained hypochlorhydria (gastric pH>4.0). Thus the biochemical indices in this group are consistent with absence of corpus atrophy. The two subjects who were seronegative for *H. pylori* were histologically normal, with normal gastrin (60 ng $l^{-1}$), PGA/C (>5.0) and low gastric pH (<2.0).

### *Precision of PGA and* H. pylori *serology for prediction of corpus atrophy*

A series of calculations of sensitivity and specificity were undertaken on the subgroup results for various levels of serum PGA alone or in combination with *H. pylori* seropositivity. A combination of PGA <80 ng $ml^{-1}$ and *H. pylori* seropositivity provided the best option in terms of both sensitivity and specificity. This combination of biomarkers predicts the presence of corpus atrophy with sensitivity 88.9% and specificity 92.3% (Table I). Using these criteria, one of nine subjects with corpus atrophy was falsely classified as negative (11.1%). This subject had PGA levels >150 ng $ml^{-1}$ but only had mild (grade 1) corpus atrophy. Three of 39 subjects without corpus atrophy were falsely classified as positive (7.7%) and, although they did not have corpus atrophy, two of them did in fact have some upper gastrointestinal tract pathology: antral atrophy and oesophageal ulcer. Only one had a completely normal mucosa.

### *Application of the chosen 'screening' criteria to the original study sample*

In our study sample, data on both serum PGA and *H. pylori* serology were available for 497 men. Of these, 96 (19.3%) were both seropositive for *H. pylori* and had PGA <80 ng $ml^{-1}$ and so would have been selected as having corpus atrophy by the screening test. Of these 96, 27.3% would be expected to be false positives ($n=26$). Further extrapolation from our subsample suggests that about a third of the 26 false positives ($n=9$) would have a normal mucosa and the rest ($n=17$) would have some other upper gastrointestinal tract pathology (although no corpus atrophy). In 75% of the 96 men selected, corpus atrophy may have been accompanied by hypochlorhydria and hypergastrinaemia. Of the 401 men who would have been cleared as not having corpus atrophy, 2.7% ($n=11$) would have been wrongly excluded from the follow-up group and in fact would be expected to have corpus atrophy.

### *Second screening stage*

Previous studies have indicated that use of the PGA/C ratio significantly improves the validity of serum pepsinogen as a screening test (Miki *et al.*, 1987, 1993; Westerveld *et al.*, 1987; Samloff, 1989). Based on these data we added a second screening stage of PGA/C ratio <2.5 to the subgroup. This resulted in detection of corpus atrophy with maximum specificity (100%) but lower sensitivity (77.8%) (Table II) due to the loss of one subject with corpus atrophy, falsely classified as negative. This subject however, had only mild (grade 1) atrophy without hypochlorhydria or hypergastrinaemia. It is likely that if the severity of his disease increased, a repeat screen, say, 5 years later, would detect his atrophy due to reduced PGA and A/C ratio. Application of this second stage to our selected group of 96 men would have resulted in 11 (2.2% of the original sample of 497 men) remaining in a group requiring invasive investigation. In total, 14 of the 85 men excluded at this stage would have been false negatives for corpus atrophy. Thus, in total, over the two stages 25 (5.1% of 486) would have been wrongly excluded, although as discussed above, it is likely that these would have been detected at a later screening if the severity of their disease had increased. The assay cost per case of corpus atrophy detected would have been approximately £500. On the basis of our data using a two-stage screening programme in males employed in an industrial setting in Stoke-on-Trent (an area with rates of gastric cancer over 30% higher than the national average), 2.2% (22 per 1000) of those screened would require further investigation (by endoscopy and biopsy). This figure is of the same order as screen detected lesions in mammography (50–60 per 1000).

### Discussion

The results of this study indicate that use of PGA levels alone is not a reliable enough indicator of the presence of gastritis and corpus atrophy in a non-patient population; of the six men with very low PGA levels (<25 ng $ml^{-1}$), one had normal mucosa and of 14 men with very high levels (>150 ng $ml^{-1}$), two (14.3%) had normal mucosa. Of those with 'normal' PGA levels between these two extremes, nine men (26.5%) had chronic gastritis, some with antral or corpus atrophy. Combining PGA levels with *H. pylori* serology greatly increased the precision with which those with significant upper gastrointestinal pathology were detected. The addition of PGC levels for the calculation of PGA/C ratio, would reduce the number of subjects referred for invasive and expensive investigation but with reduced sensitivity in terms of cases of corpus atrophy detected. Those selected, however, would be more likely to have corpus atrophy accompanied by hypochlorhydria, the 'classic' scenario for increased risk of gastric carcinoma.

There was clearly an association between the pattern of gastritis and PGA levels, with the proportion of corpus predominant gastritis decreasing and of pangastritis and antral predominant gastritis increasing with increasing PGA levels. This non-invasive technique appears, therefore, to

**Table I** 2×2 contingency table for prediction of corpus atrophy by serological tests: PGA, <80 ng $ml^{-1}$ and *H. pylori* seropositivity

| | *Histology* | |
|---|---|---|
| *Serology* | *Corpus atrophy* | *No corpus atrophy* |
| Corpus atrophy | 8 | 3 |
| No corpus atrophy | 1 | 36 |

**Table II** 2×2 contingency table for prediction of corpus atrophy by serological tests: PGA<80 ng $ml^{-1}$, PGA/C<2.5 and *H. pylori* seropositivity

| | *Histology* | |
|---|---|---|
| *Serology* | *Corpus atrophy* | *No corpus atrophy* |
| Corpus atrophy | 7 | 0 |
| No corpus atrophy | 2 | 39 |

provide valuable information about the overall spectrum of gastritis in *H. pylori* seropositive people and would allow different patterns of gastritis to be studied in different population groups. In addition, factors affecting the progression of gastritis to antrum or corpus predominant patterns could be investigated.

Ulcers were seen at endoscopy in only 2 of the 54 subjects endoscoped. One with a duodenal ulcer had antrum predominant *H. pylori* gastritis on histology and was seropositive with a PGA of 114.4 ng $ml^{-1}$. The second had an oesophageal ulcer, was weakly seropositive for *H. pylori* but histologically had normal gastric mucosa. He was also hypochlorhydric (pH 6.2) but did not have raised serum gastrin.

*H pylori*-associated gastritis is therefore a heterogenous condition histologically. Some individuals have an antrum predominant pattern of disease; these may develop duodenal ulceration but have a reduced risk of developing gastric cancer (Lee *et al*., 1990). Others have pangastritis or corpus predominant gastritis with progressive mucosal atrophy and are at increased risk of developing gastric carcinoma. The precise factors that determine the topographical pattern or progression of the gastritis are not currently known.

The 26 cases of gastritis in the endoscoped group included 20 associated with *H. pylori* and six of 'special forms' of gastritis; five of lymphocytic gastritis and one of *Gastrospirillum hominis* infection. Surprisingly, no examples of reactive gastritis were detected, in contrast to a frequency of about 10% of endoscopic gastric biopsies from routinely endoscoped symptomatic patients seen by the same pathologist. This suggests that reactive gastritis is uncommon in the healthy population and thus may be symptomatic in a high proportion of cases. Lymphocytic gastritis is a distinctive condition histologically, which, in our experience, is typically *H. pylori* negative on histology but positive on serology. It is seen in up to 4% of biopsies from endoscoped patients with gastritis (Dixon *et al*., 1988) and so the finding of this pattern in 5/26 (19.2%) patients with gastritis in this study is surprisingly high. However, three of these five were among the Group 1 patients with low PGA and corpus atrophy, which explains their selection for endoscopy. Surprisingly, subjects with lymphocytic gastritis tended to be younger (median age 34, four of five aged $<40$ years) than other seropositive subjects with gastritis (median age 48, 5/20 aged $<40$ years). Three of four with adequately deep corpus biopsies showed a degree of atrophy, supported by hypochlorhydria and high serum gastrin, suggesting that lymphocytic gastritis may have a high frequency of progression to corpus atrophy at a relatively young age. Atrophic corpus mucosa and hypochlorhydria are well known to be associated with increased risk of gastric carcinoma. Griffiths *et al*. (1994) recently found a frequency of lymphocytic gastritis of 12% in non-neoplastic mucosa in patients who had gastric adenocarcinoma and in those with B-cell lymphoma. Clearly, the significance of lymphocytic gastritis as a possible premalignant condition warrants further study. Weak serological cross-reactions with *H. pylori* antigens have previously been recognised in patients with *Gastrospirillum hominis* infection, which is estimated to account for about 0.2% cases of chronic gastritis (Heilmann and Borchard, 1991).

### Conclusion

Pepsinogen A and C levels and A/C ratio are non-invasive markers for gastritis of value in both epidemiological and clinical studies. Our results indicate that their use in combination with *H. pylori* serology could form the basis of a biomarker strategy likely to prove useful in high-risk population groups, such as first-degree relatives of gastric cancer cases (Graham and Lilienfield, 1958; Langman, 1988) or those in occupational groups traditionally associated with an increased risk of gastric cancer. Gastric carcinoma is more common in men than women. The study was conducted amongst a male population and therefore the results may not be applicable to a female population. The results of this work build upon, and are consistent with, data from studies conducted within different populations around the world. Further research is now needed to test the validity of the proposed test in women and to evaluate the proposed screening programme in terms of feasibility and effectiveness in target populations.

**Acknowledgements**

This research was funded by grants to T Knight and JB Elder from the West Midlands Regional Health Authority, North Staffordshire Health Authority and Medical Institute and by the Imperial Cancer Research Fund. It would not have been possible without the heroic contributions of the volunteers nor the patience of the staff of the North Staffordshire Gastroenterology Department. Our thanks are also extended to those who helped with data collection in the factories and to Dr Caroline Musgrove and staff of the North Staffordshire Pathology Department and other assay laboratories in Dusseldorf, Belfast, Stoke, Oxford and Weybridge.

### References

ARDILL J. (1979). Radioimmunoassay of G.I. hormones. *Clin. Endocrinol. Metab*., **8**, 265–280.

BRANDT-RAUF PW. (1987). Occupational cancer and carcinogenesis. *Occupational Medicine*, **2**, 123–133.

COGGON D, BARKER DJP AND COLE RB. (1990). Stomach cancer and work in dusty industries. *Br. J. Ind. Med*., **47**, 298–301.

CORREA P. (1988). A human model of gastric carcinogenesis. *Cancer Res*., **48**, 3554–3560.

DIXON MF. (1992). *Helicobacter pylori* and chronic gastritis. In Helicobacter pylori *and gastroduodenal disease*. 2nd edn. Rathborne BJ, Heatley RV. (eds). pp. 124–139. Blackwell Scientific: Oxford.

DIXON MF. (1994). Recent advances in gastritis. *Curr. Diagn. Pathol*., **1**, 80–89.

DIXON MF, WYATT JI, BURKE DA AND RATHBONE BJ. (1988). Lymphocytic gastritis–relationship with *Campylobacter pylori* infection. *J. Pathol*., **154**, 125–132.

GRAHAM S AND LILIENFIELD AM. (1958). Genetic studies of gastric cancer in humans. *Cancer*, **11**, 945–958.

GRIFFITHS AP, WYATT JI, JACK AS AND DIXON MF. (1994). Lymphocytic gastritis, gastric adenocarcinoma and primary gastric lymphoma. *J. Clin. Pathol*., **47**, 1123–1124.

HEILMANN KL AND BORCHARD F. (1991). Gastritis due to spiral-shaped bacteria other than *Helicobacter pylori*, clinical, histological and ultrastructural findings. *Gut*, **32**, 137–140.

HENGELS KJ AND STROHMEYER G. (1989). Pepsinogens A and C: purification from human gastric mucosa and determination in serum by optimised radioimmunoassays. *Zeitung Gastroenterologie*, **27**, 406–411.

JASS JR. (1980). Role of intestinal metaplasia in the histogenesis of gastric carcinoma. *J. Clin. Pathol*., **33**, 801–810.

KEKKI M, SAMLOFF IM, VARIS K AND IHAMAKI T. (1991). Serum pepsinogen I and serum gastrin in the screening of severe atrophic corpus gastritis. *Scand. J. Gastroenterol*., **26**, 109–116.

KNIGHT T, GREAVES S, WILSON A, HENGELS K, NEWELL D, CORLETT M, WEBB P, FORMAN D AND ELDER J. (1995). Variability in serum pepsinogen levels in an asymptomatic population. *Eur. J. Gastroenterol. Hepatol*. **7**, in press.

LANGMAN MJS. (1988). Genetic influences upon gastric cancer frequency. In *Gastric Carcinogenesis*, Reed PI and Hill MJ (eds). pp. 81–86. Excerpta Medica: Oxford.

LEE S, IIDA M, YAO T, SHINDS S, NOSE Y, AKAZAWA K, OKABE H AND FUJISHIMA M. (1990). Risk of gastric cancer in patients with non-surgically treated peptic ulcer. *Scand. J. Gastroenterol*., **25**, 1223–1226.

MIKI K, ICHINOSE M, SHIMIZU A, HUANG SC, OKA H, FURIHATA C, MATSHUSHIMA T AND TAKAHASHI K. (1987). Serum pepsinogens as a screening test of extensive chronic gastritis. *Gastroenterologica Japonica*, **22**, 133–141.

MIKI K, ICHINOSE M, ISHIKAWA KB, YAHAGI N, MATSUSHIMA M, KAKEI N, TSUKADA S, KIDO M, ISHIAMA S, SHIMIZU Y, SUZUKI T AND KUROKAWA K. (1993). Clinical application of serum pepsinogen I and II levels for mass screening to detect gastric cancer. *Japan. J. Cancer Res.*, **84**, 1086–1090.

PRICE AB. (1991). The Sydney system: histological division. *J. Gastroenterol. Hepatol.*, **6**, 209–222.

SAMLOFF IM. (1989). Peptic ulcer: the many proteases of aggression. *Gastroenterology*, **96**, 586–595.

SAMLOFF IM, VARIS K, IHAMAKI T, SIURALA M AND ROTTER JI. (1982). Relationships among serum pepsinogen I, serum pepsinogen II and gastric mucosal histology. *Gastroenterology*, **83**, 204–209.

SIPPONEN P. (1991). Long term consequences of gastroduodenal inflammation. *Eur. J. Gastroenterol. Hepatol.*, **44**, 525–529.

SIPPONEN P, KEKKI M, HAAPAKOSHI J, IHAMAKI T, SIURALA M. (1985). Gastric cancer risk in chronic atrophic gastritis: statistical calculations of cross-sectional data. *Int. J. Cancer*, **35**, 173–177.

SITAS F, SMALLWOOD R, JEWELL D, MILLARD PR, NEWELL DG, MEUWISSEN SGM, MOSES S, ZWIES A AND FORMAN D. (1993). Serum anti-*Helicobacter pylori* IgG antibodies and pepsinogens A and C as serological markers of chronic atrophic gastritis. *Cancer Epidemiol., Biomarkers and Prevention*, **2**, 119–123.

SORAHAN T, PARKES HG, VEYS C, WATERHOUSE JAH, STRANGHAN JK AND NUTT A. (1989). Mortality in the British rubber industry. *Br. J. Ind. Med.*, **46**, 1–11.

STEER HW, HAWTIN PR AND NEWELL DG. (1987). An Elisa technique for the serodiagnosis of *Campylobacter pyloridis* infection in patients with gastritis and benign duodenal ulceration. *Serodiagnosis and Immunotherapy*, **1**, 253–259.

TALLEY NY, NEWELL DG, ORMOND JE, CARPENTER HE, WILSON WE, ZINSMEISTER AR, PEREZ-PEREZ GI AND BLASER MJ. (1991). Serodiagnosis of *Helicobacter pylori*: comparison of enzyme-linked immunosorbent assays. *J. Clin. Microbiol.*, **29**, 1635–1639.

VARIS K. (1983). Surveillance of pernicious anaemia. In *Precancerous Lesions of the Gastrointestinal Tract*, Sherlock P, Morson BC, Barbara L and Veronese U (eds). pp. 189–194. Raven Press: New York.

VARIS K, SAMLOFF IM, IHAMAKI T AND SIURALA M. (1979). An appraisal of tests for severe atrophic gastritis in relatives of patients with pernicious anaemia. *Dig. Dis. Sci.*, **24**, 187–191.

VARIS K, KEKKI M, HAKKINON M, SIPPONEN P AND SAMLOFF IM. (1991). Serum pepsinogen I and serum gastrin in the screening of atrophic pangastritis with high risk of gastric cancer. *Scand. J. Gastroenterol.*, **26**, 117–123.

VEYS CA. (1985). Stomach cancer in Stoke-on-Trent is excessive: are the dusty trades implicated? *Hum. Toxicol.*, **4**, 100.

WEBB PM, KNIGHT TM, GREAVES S, WILSON A, NEWELL DG, ELDER JB AND FORMAN D. (1994). Relation between infection with *Helicobacter pylori* and living conditions in childhood: evidence for person to person transmission in early life. *Br. Med. J.*, **308**, 750–753.

WESTERVELD BD, PALS G, LAMERS BHW, DEFIZE J, PRONK JC, FRANTS RR, OOMS CM, KREUNIG J, KOSTENSE PJ, ERIKSSON AW AND MEUWISSEN SGM. (1987). Clinical significance of pepsinogen A, isozymogens, serum pepsinogen A and C levels and serum gastrin levels. *Cancer*, **59**, 952–958.

WYATT JI. (1995). Histopathology of gastroduodenal inflammation; the impact of *Helicobacter pylori*. *Histopathology*, **26**, 1–15.